# *ABCA1* missense mutations are associated with Lewy body dementia

**DOI:** 10.1002/alz.092678

**Published:** 2025-01-03

**Authors:** Anindita Ray, Paolo Reho, Giancarlo Logroscino, Sami Barmada, Owen A. Ross, Sonja W Scholz, International LBD Genomics Consortium

**Affiliations:** ^1^ National Institute of Neurological Disorders and Stroke, Bethesda, MD USA; ^2^ Laboratory of Precision Environmental Health, Mailman School of Public Health, Columbia University, New York, NY USA; ^3^ University of Bari, Bari Italy; ^4^ Michigan Alzheimer’s Disease Center, Ann Arbor, MI USA; ^5^ Mayo Clinic, Jacksonville, FL USA; ^6^ Johns Hopkins University Medical Center, Baltimore, MD USA

## Abstract

**Background:**

Lewy body dementia (LBD) is the second most prevalent dementia in the United States after Alzheimer’s disease (AD). Recent studies have implicated rare mutations in two lipid transport genes, *ABCA1* and *ATP8B4*, in Alzheimer’s disease. Substantial co‐pathology and shared risk factors indicate an intersectional genetic architecture between LBD and AD; therefore, we investigated the association of rare mutations in *ABCA1* and *ATP8B4* with LBD. Additionally, we examined rare mutations in 25 genes involved in *ABCA1*‐related lipid‐specific pathway with risk of developing LBD.

**Method:**

We annotated rare missense and loss‐of‐function (LOF) mutations in *ABCA1* and *ATP8B4* genes, using Ensembl Variant Effect Predictor, in previously published whole‐genome sequence data from 2,591 LBD cases and 4,027 healthy controls of European ancestry. To test the association with LBD risk, we applied the optimized sequence kernel association test (SKAT‐O), followed by single‐variant association test (Fisher’s exact test), and multiple testing (Bonferroni correction). Additionally, we evaluated rare coding variants of 25 genes in *ABCA1*‐related positive regulation of the cholesterol efflux pathway (GO: 0010875) by performing single‐gene burden and gene‐set burden analyses.

**Results:**

We found a significant enrichment of rare *ABCA1* missense mutations in LBD (SKAT‐O p = 2.68E‐02). However, no significant associations were found between LBD and *ABCA1* LOF, and *ATP8B4* LOF or missense mutations. Interestingly, the single‐variant analysis identified two *ABCA1* missense variants, *c.5398A>C*; p.N1800H (Fisher’s test: p‐value = 1.16E‐04) and *c.3544G>A*; p.A1182T (p‐value = 4.29E‐02), with the p.N1800H substitution surviving Bonferroni multiple testing. The p.N1800H variant is reported to be pathogenic, and it was observed in twelve LBD patients and one control, with an odds ratio of 18.48 (95%CI = 2.4‐142.8). This mutation is reported to be pathogenic, in our LBD cohort, six out of twelve *ABCA1* p.N1800H carriers also carry the *APOE* ε4 risk allele. However, neither a single‐gene burden nor a gene‐set burden analysis in 25 genes in the positive regulation of the cholesterol efflux pathway revealed any significant associations with LBD.

**Conclusion:**

Our findings reveal significant enrichment of *ABCA1* missense mutations in LBD emphasizing the genomic complexity surrounding LBD and AD.

